# [^11^C]PK11195 binding in Alzheimer disease and progressive supranuclear palsy

**DOI:** 10.1212/WNL.0000000000005610

**Published:** 2018-05-29

**Authors:** Luca Passamonti, Patricia Vázquez Rodríguez, Young T. Hong, Kieren S.J. Allinson, W. Richard Bevan-Jones, David Williamson, P. Simon Jones, Robert Arnold, Robin J. Borchert, Ajenthan Surendranathan, Elijah Mak, Li Su, Tim D. Fryer, Franklin I. Aigbirhio, John T. O'Brien, James B. Rowe

**Affiliations:** From the Departments of Clinical Neurosciences (L.P., P.V.R., Y.T.H., P.S.J., R.J.B., T.D.F., F.I.A., J.B.R.) and Psychiatry (W.R.B.-J., R.A., A.S., E.M., L.S., J.T.O.), University of Cambridge, UK; Istituto di Bioimmagini e Fisiologia Molecolare (L.P.), Consiglio Nazionale delle Ricerche, Milano, Italy; Wolfson Brain Imaging Centre (Y.T.H., D.W., T.D.F., F.I.A.), University of Cambridge; Department of Histopathology (K.S.J.A.), Cambridge University Hospitals NHS Foundation Trust, Cambridge, UK; China–UK Centre for Cognition and Ageing Research (L.S.), Southwest University, Chongqing, China; and Medical Research Council Cognition and Brain Sciences Unit (J.B.R.), UK.

## Abstract

**Objective:**

We tested whether in vivo neuroinflammation relates to the distinctive distributions of pathology in Alzheimer disease (AD) and progressive supranuclear palsy (PSP).

**Methods:**

Sixteen patients with symptomatic AD (including amnestic mild cognitive impairment with amyloid-positive PET scan), 16 patients with PSP–Richardson syndrome, and 13 age-, sex-, and education-matched healthy controls were included in this case-control study. Participants underwent [^11^C]PK11195 PET scanning, which was used as an in vivo index of neuroinflammation.

**Results:**

[^11^C]PK11195 binding in the medial temporal lobe and occipital, temporal, and parietal cortices was increased in patients with AD, relative both to patients with PSP and to controls. Compared to controls, patients with PSP showed elevated [^11^C]PK11195 binding in the thalamus, putamen, and pallidum. [^11^C]PK11195 binding in the cuneus/precuneus correlated with episodic memory impairment in AD, while [^11^C]PK11195 binding in the pallidum, midbrain, and pons correlated with disease severity in PSP.

**Conclusions:**

Together, our results suggest that neuroinflammation has an important pathogenic role in the 2 very different human neurodegenerative disorders of AD and PSP. The increase and distribution of microglial activation suggest that immunotherapeutic strategies may be useful in slowing the progression of both diseases.

There is evidence that microglia show increased activation in Alzheimer disease (AD), Parkinson disease, Huntington disease, and progressive supranuclear palsy (PSP).^1–12^ Furthermore, genetic association studies in AD reveal variations in genes that contribute to immune signaling as risk factors.^13^ This raises the possibility of immune-therapeutic strategies for prevention and disease modification.

However, key issues need to be addressed before such strategies can be applied, including the confirmation of clinicopathologic correlations of neuroinflammation and the establishment of the potential utility of biomarkers for measuring and tracking neuroinflammation in vivo. Despite the importance of neuroinflammation, there is still insufficient information regarding the extent and regional distribution of microglial activation in patients with neurodegenerative conditions, and their association with clinical markers of disease severity or their relationship to systemic inflammatory markers.

[^11^C]PK11195 is a well-established PET marker of in vivo microglial activation,^1,2,6–8,10,14^ although microglial activation represents only part of the complex cascade of events in neuroinflammation.^15^ Here, we assessed the magnitude and patterns of [^11^C]PK11195 binding in 2 very different neurodegenerative entities, AD and PSP, characterized by distinct anatomical distributions of pathology. The value of this comparison does not lie in the differential diagnosis between these clinically diverse entities but rather in establishing the distribution of neuroinflammation in 2 distinct tauopathies. We tested whether [^11^C]PK11195 binding relates to the distinctive distributions of pathology in typical amnestic AD and PSP–Richardson syndrome, and whether [^11^C]PK11195 binding relates to different measures of clinical severity in AD and PSP.

## Methods

### Participants

The current study was conducted within the context of the NIMROD (Neuroimaging of Inflammation in Memory and Related Other Disorders) Study.^16^ We recruited 16 PSP patients with probable PSP by the 1996 Movement Disorder Society criteria (representing a “classic phenotype,” which is sometimes referred to as Richardson syndrome), but all patients also met 2017 revised criteria for probable PSP–Richardson syndrome^17,18^; 9 patients met diagnostic criteria for probable AD^19^ (typical amnestic phenotype, without biomarkers) and 7 patients had amnestic mild cognitive impairment (MCI). The patients with amnestic MCI had (1) a Mini-Mental State Examination (MMSE) score of >24/30, (2) memory impairment at least 1.5 SDs below that expected for age and education,^20^ and (3) biomarker evidence of amyloid pathology (positive Pittsburgh compound B [PiB]-PET scan) (MCI+). Thirteen age-, sex-, and education-matched healthy controls with no history of major psychiatric or neurologic illnesses, head injury, or any other significant medical comorbidity were also recruited. All participants were older than 50 years, had sufficient proficiency in English for cognitive testing, did not have any acute infectious or symptomatic systemic inflammatory disorder (e.g., lupus, rheumatoid arthritis, Crohn disease, polymyalgia rheumatica), and had no contraindications to MRI. Patients were identified from the specialist clinics at the Cambridge University Hospitals NHS Trust and the Dementias and Neurodegenerative Diseases Research Network, while healthy controls were recruited via the Dementias and Neurodegenerative Diseases Research Network, which is part of the National Institute for Health Research (NIHR) Clinical Research Network (nihr.ac.uk/nihr-in-your-area/dementias-and-neurodegeneration/).

### Standard protocol approvals, registrations, and patient consents

All participants had mental capacity, and we obtained informed written consent from patients (as principal participants) and patients' designated informants (for providing informant information) in accordance with the Declaration of Helsinki. The study was approved by the local ethics committee.

### Clinical, cognitive, and blood assessment

Participants' assessment included clinical indices of disease severity, such as Rey Auditory Verbal Learning Test (RAVLT) in AD/MCI+ patients and Progressive Supranuclear Palsy Rating Scale (PSPRS) in patients with PSP.^21^ Demographic measures and neuropsychological tests (i.e., MMSE and Addenbrooke's Cognitive Examination–Revised) as well as a blood sample to assess the levels of 3 basic peripheral markers of inflammation (i.e., C-reactive protein [CRP], erythrocyte sedimentation rate [ESR], and white blood cell count) were also obtained from all participants. The CRP, ESR, and white blood cell biomarkers were included on the basis that peripheral inflammation may facilitate the development of neuroinflammation and neurodegeneration,^22–24^ and such peripheral markers might augment the monitoring of immunotherapeutic trials if related to central inflammation.

### Neuroimaging assessment

All participants underwent MRI on a 3T Siemens Magnetom Tim Trio or Verio scanner (medical.siemens.com) using a magnetization-prepared rapid-acquisition gradient echo T1-weighted sequence. The T1-weighted sequence (repetition time = 2,300 milliseconds, echo time = 2.98 milliseconds, field of view = 240 × 256 mm^2^, 176 slices of 1-mm thickness, flip angle = 9°) was used to facilitate tissue class segmentation (gray and white matter, together with CSF) and to allow nonrigid registration of standard space regions of interest (ROIs) to subject MRI space (using a modified version of the Hammers atlas, which included the midbrain, pons, cerebellar gray matter, and dentate nucleus of the cerebellum ROIs). Each T1 image was nonrigidly registered to the ICBM2009a template brain using ANTS (picsl.upenn.edu/ANTS/), and the inverse transform was applied to the modified Hammers atlas (resliced from MNI152 to ICBM2009a space) to bring the ROIs to subject MRI space.

All participants underwent [^11^C]PK11195 PET imaging for assessment of the extent and distribution of brain inflammation. [^11^C]PK11195 and [^11^C]PiB were produced with high radiochemical purity (>95%), with [^11^C]PiB having a specific activity >150 GBq/μmol at the end of synthesis, while [^11^C]PK11195 specific activity was around 85 GBq/μmol at the end of synthesis. PET scanning was performed with a GE Advance PET scanner (GE Healthcare, Waukesha, WI) and a GE Discovery 690 PET/CT, with attenuation correction provided by a 15-minute ^68^Ge/^68^Ga transmission scan and a low-dose CT scan, respectively. The emission protocols were 550 MBq [^11^C]PiB injection followed by imaging from 40 to 70 minutes post injection, and 75 minutes of dynamic imaging (55 frames) starting concurrently with a 500-MBq [^11^C]PK11195 injection. Each emission frame was reconstructed using the PROMIS 3-dimensional filtered back projection algorithm into a 128 × 128 matrix 30-cm transaxial field of view, with a transaxial Hann filter cutoff at the Nyquist frequency.^25^ Corrections were applied for randoms, dead time, normalization, scatter, attenuation, and sensitivity. Each emission image series was aligned using SPM8 to reduce the effect of patient motion during data acquisition (fil.ion.ucl.ac.uk).

The mean aligned PET image (and hence the corresponding aligned PET image series) was rigidly registered to the T1-weighted MRI. For [^11^C]PiB, we used reference tissue ROI defined by ≥90% on the SPM8 gray matter probability map (smoothed to PET resolution) in the superior cerebellar cortex.^26^ For [^11^C]PK11195, supervised cluster analysis was used to determine the reference tissue time-activity curve.^27^ All ROI data were corrected for CSF contamination through division with the mean ROI probability (normalized to 1) of gray + white matter, using SPM8 probability maps smoothed to PET resolution. To test whether correction for CSF affected the main results, we repeated all the [^11^C]PK11195 ROI PET analyses using data not corrected for CSF contamination (see PET statistical analyses and results sections).

[^11^C]PiB data were quantified using standardized uptake value ratio by dividing the mean CSF-corrected radioactivity concentration in each Hammers atlas ROI by the corresponding mean CSF-corrected radioactivity concentration in the reference tissue ROI. For [^11^C]PK11195, nondisplaceable binding potential (BP_ND_), a measure of specific binding, was determined for each ROI using a basis function implementation of the simplified reference tissue model, both with and without CSF contamination correction.^28^ [^11^C]PK11195 BP_ND_ maps were also generated using this basis function simplified reference tissue model approach. [^11^C]PiB data were treated as dichotomous measures (i.e., positive or negative) and considered positive if the average standardized uptake value ratio across the cortical ROIs was >1.5.^29^

To compare [^11^C]PK11195 binding across groups (AD/MCI PiB+, PSP, and controls), individual ROI BP_ND_ values for [^11^C]PK11195 were used in a repeated-measures general linear model to test for the main effect of ROI, main effect of group, and group × ROI interaction. Age and sex were included as covariates of no interest. For the AD/MCI+ and PSP groups, we also tested Pearson correlations between regional [^11^C]PK11195 BP_ND_ and disease severity using the RAVLT scores for AD/MCI+ patients and the PSPRS for patients with PSP. Finally, we tested for associations between neuroinflammation and peripheral markers of inflammation using Pearson correlations.

### Data availability

Anonymized data will be shared by request from any qualified investigator.

## Results

### Clinical, cognitive, and blood findings

The patient and control groups were matched for age, sex, and education (table). Nevertheless, to account for any possible residual confounding effect associated with variability in demographic measures, age and sex were included as covariates of no interest in the general linear models of the main effect of ROI, the main effect of group, and the group × ROI interaction. As expected, there was a significant main effect of group for cognitive measures, driven by reduced MMSE and Addenbrooke's Cognitive Examination–Revised scores in AD/MCI+ and PSP patients relative to healthy controls (table). Episodic memory, as assessed via the RAVLT (delayed recall), was significantly impaired in AD/MCI+ patients relative to controls (table). Although none of the participants included had acute inflammatory conditions (see exclusion criteria), patients with PSP displayed higher CRP levels than AD/MCI+ patients and controls, despite normal leukocyte count and ESR (table).

**Table:**
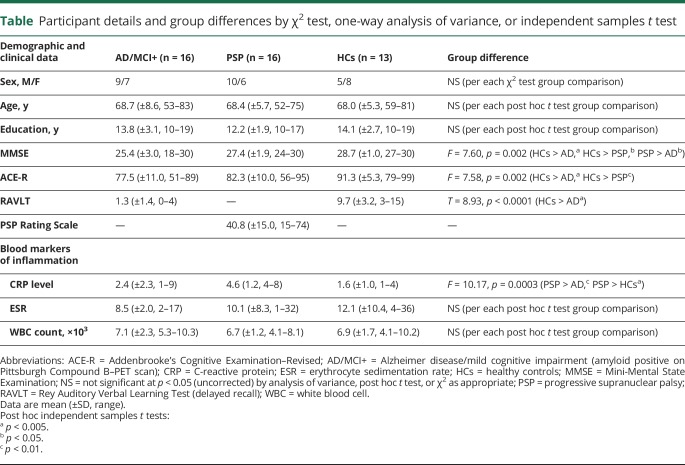
Participant details and group differences by χ^2^ test, one-way analysis of variance, or independent samples *t* test

### Neuroimaging findings

**Figure 1:**
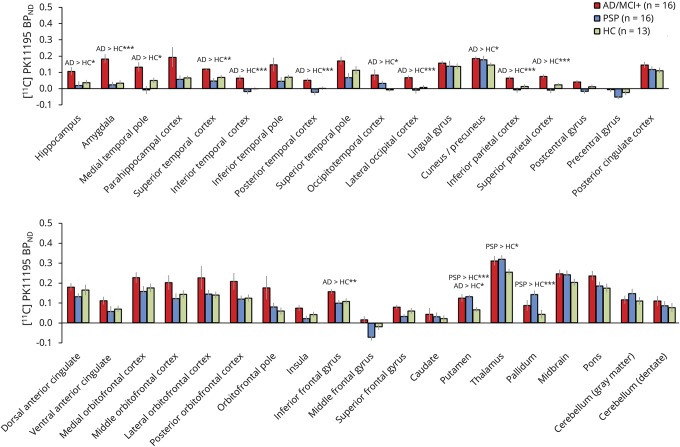
[^11^C]PK11195 binding in AD, PSP, and HCs The bar plots represent the mean values (± SE) of the [^11^C]PK11195 BP_ND_ in each region of interest for the participant groups: AD and MCI+, PSP, and HCs. The [^11^C]PK11195 BP_ND_ data reported here are corrected for CSF contamination. See the results section for statistics related to CSF-corrected and uncorrected data. Post hoc *t* tests: *p < 0.05, **p < 0.01, ***p < 0.005. AD = Alzheimer disease; BP_ND_ = nondisplaceable binding potential; HC = healthy control; MCI+ = amyloid-positive mild cognitive impairment; PSP = progressive supranuclear palsy.

In the repeated-measures analysis of regional binding, we found a significant main effect of ROI (*F*_2,36_ = 3.8, *p* < 0.001), main effect of group (*F*_2,36_ = 5.7, *p* < 0.006), and a group × ROI interaction (*F*_2,70_ = 2.6, *p* < 0.001) (figure 1). The group and interaction effects were driven in part by higher [^11^C]PK11195 BP_ND_ values in the AD/MCI+ group relative to both the PSP and control groups, in cortical and subcortical ROIs, including occipital, parietal, and temporal cortices, as well as in the hippocampus, amygdala, and other medial temporal lobe ROIs (figure 1). The PSP group, relative to controls, showed increased [^11^C]PK11195 BP_ND_ in the thalamus, putamen, and pallidum (figure 1).

**Figure 2:**
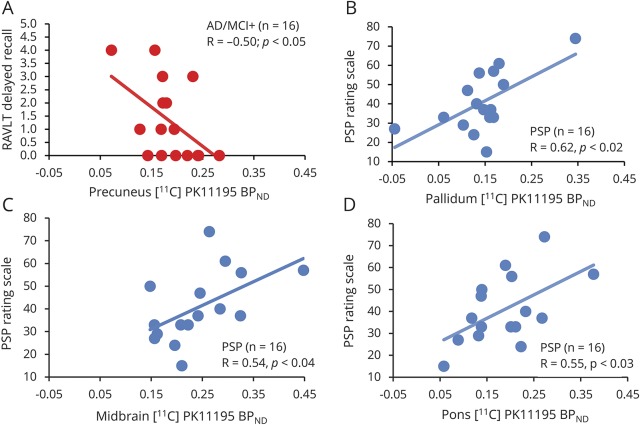
[^11^C]PK11195 binding correlates with clinical severity in AD and PSP (A) Correlation between [^11^C]PK11195 BP_ND_ values in the precuneus (x-axis) and RAVLT scores (y-axis) in patients with AD and MCI+ (red dots). (B–D) Correlation between [^11^C]PK11195 BP_ND_ values in the pallidum, midbrain, and pons (x-axes) and PSP Rating Scale (y-axes) in patients with PSP. AD = Alzheimer disease; BP_ND_ = nondisplaceable binding potential; MCI+ = amyloid-positive mild cognitive impairment; PSP = progressive supranuclear palsy; RAVLT = Rey Auditory Verbal Learning Test.

Repeating these analyses using ROI [^11^C]PK11195 BP_ND_ values that were not corrected for CSF partial volume effects yielded similar results (*F*_2,36_ = 2.2, *p* < 0.0001 for the main effect of ROIs; *F*_2,36_ = 6.1, *p* < 0.006 for the main effect of group; and *F*_2,70_ = 2.0, *p* < 0.0001 for the group × ROI interaction). We then tested whether regional [^11^C]PK11195 BP_ND_ related to disease severity in each clinical group. In the AD/MCI+ group, there was a significant *negative* correlation between the RAVLT scores (delayed recall at 30 minutes) and [^11^C]PK11195 BP_ND_ in the precuneus (figure 2A). In the PSP group, we found a significant *positive* correlation for [^11^C]PK11195 BP_ND_ in the pallidum, midbrain, and pons and disease severity, as assessed via the PSPRS (figure 2, B–D).

## Discussion

The brain regions with the most marked abnormalities of [^11^C]PK11195 binding in AD/MCI+ and PSP groups were those predicted from the established distribution of neurodegeneration of each disease. Specifically, patients with amnestic AD/MCI+ had evidence of increased neuroinflammation in the medial temporal lobe as well as parietal and lateral temporal cortices.^30–33^ Conversely, patients with PSP had evidence of enhanced neuroinflammation in the thalamus, pallidum, and putamen, a group of subcortical regions that have been implicated in the pathophysiology of PSP.^34,35^ The increased [^11^C]PK11195 binding in the basal ganglia in PSP is also consistent with preliminary findings reported in a study with 4 patients with PSP.^10^

Our data demonstrate that the density and distribution of activated microglia in living patients with AD and PSP mirror the typical neuropathologic changes characteristic of each disorder. This could result from a causal link between neuroinflammation and neurodegeneration, although the association might also derive from the process of neurodegeneration itself. A cross-sectional and noninterventional study such as this one cannot alone provide the direction of causality. Nevertheless, the disease-specific anatomical distributions of activated microglia in AD and PSP suggest a regional association rather than a side effect of a global increased [^11^C]PK11195 binding in response to a general inflammatory insult.

Our PET data are also in keeping with previous postmortem findings,^3^ which demonstrated that microglia burden (as assessed via LN3 immunostaining) and interleukin 1β and transforming growth factor β expression showed a disease-specific topological relationship with the pathologic hallmarks of AD and PSP.^3^ More specifically, the previous postmortem study^3^ found that patients with AD had significantly higher microglia density and interleukin 1β expression in the parietal cortices compared to patients with PSP and controls, while the microglia density and cytokine expression was greater in the substantia nigra of patients with PSP relative to patients with AD and controls.^3^ The expression of transforming growth factor β was also increased in frontal and parietal cortices in patients with AD relative to patients with PSP or controls.^3^ Together with our findings, these data suggest that microglia activation and cytokine expression coexist with the pathogenic processes underlying AD and PSP and could contribute to the process of ongoing neurodegeneration.^3^ If so, this would warrant further investigation of immune-therapeutic strategies to modulate neuroinflammation in AD and PSP, although evidence from earlier anti-inflammatory trials in AD remains controversial,^36,37^ and no such clinical trials have been conducted in PSP.

Our data also confirmed the hypothesis that [^11^C]PK11195 binding correlates with disease severity in both AD/MCI+ and PSP; more specifically, with severity of episodic memory impairment as assessed via the RAVLT in AD/MCI+, and with PSP severity as measured via the PSPRS in PSP. Again, these effects were not global correlations but adhered to the functional anatomy of cognitive and motor symptoms in AD and PSP (i.e., cuneus/precuneus in relation to episodic memory deficits in AD as well as the pallidum, midbrain, and pons in relation to PSPRS in PSP).

Despite that a symptomatic acute infection and chronic extraneural inflammatory condition were exclusion criteria of our study, the patients with PSP showed higher levels of CRP in the blood, a common peripheral marker of inflammation. We acknowledge that the dysphagia and bladder dysfunction frequently experienced by patients with PSP can put them at risk of respiratory and urinary tract infections. However, the average CRP level in our PSP group was 4.6 (±1.2), which is well below the values expected in acute inflammatory states. In addition, the leucocyte count and ESR were both normal. The increased CRP levels in PSP might therefore reflect underlying chronic inflammatory states that sustain or even accelerate the neuroinflammation associated with PSP, in the absence of acute infection. It will be useful to replicate these findings in larger clinical cohorts and use more detailed peripheral markers of inflammation (e.g., immune-phenotyping) for characterization and classification of the immune cells in circulation in AD and PSP.

Overall, the use of [^11^C]PK11195 PET could provide helpful information to stratify patients in future clinical trials or to track the effects of treatments targeting neuroinflammation in neurodegenerative disorders such as AD and PSP. However, to fully meet its potential toward these directions, additional properties are necessary to show for this biomarker of neuroinflammation. Specifically, although recent longitudinal studies in AD have demonstrated that changes in [^11^C]PK11195 binding may be associated with disease progression,^14,38^ such a correlation has not been established in PSP. Neuroinflammation might be stable in symptomatic stages of PSP, as suggested by a pilot study of 2 patients with PSP.^10^ Furthermore, increased levels of serum neurofilament light protein have been found in both AD and PSP, and correlated with disease severity^39–41^; hence, future studies may test whether markers of neuroinflammation are associated with neurofilament light protein levels in the serum. Perhaps more importantly, it remains to be determined whether the putative effects of anti-inflammatory therapies can reduce the elevated [^11^C]PK11195 binding in AD and PSP and, consequently, could help slow the progression of these disorders. This would also enable mediation analysis to test the causality between immune-reactivity and disease progression in dementia and related disorders. Furthermore, we suggest that multitracer PET studies will be useful to formally assess how neuroinflammation relates to other important molecular aspects in dementia and related disorders including, for example, studying how neuroinflammation is associated with amyloid load in AD^14^ as well as with tau burden in AD and PSP. A cross-sectional and single-tracer study like the present one is not able to address such interesting and open questions, although it represents the necessary first step toward achieving this goal.

Technical considerations regarding the [^11^C]PK11195 BP_ND_ PET methods should also be considered. In particular, our main regional PET analyses used partial volume correction for CSF, which controlled for differences in CSF signal contamination within each region and across the different diagnostic groups (i.e., AD/MCI+, PSP, and control groups). Although this approach is important to reduce the potential influence of brain volume loss seen in AD/MCI+ and PSP, this MRI-guided method is subject to error because of imperfect registration of PET and MRIs, together with errors in segmentation and point spread function modeling. However, we note that using uncorrected PET data yielded similar results in terms of the main effect of ROI, main effect of group, and group × ROI interaction, which provides substantiation of the CSF-corrected results. The supervised cluster method for estimating [^11^C]PK11195 BP_ND_ could also have introduced an underestimation bias, as the reference tissue may have still included specific binding of the radioligand. In any case, this may have only reduced the effect sizes without altering the risk of reporting false-positive results.

We also highlight that our data are specific to [^11^C]PK11195 and do not inevitably generalize to second-generation translocator protein (TSPO) ligands (e.g., PBR28) or alternative tracers of neuroinflammation over and above those that bind to TSPO (e.g., COX-1, MPO, macrophage infiltration).^42–44^ Further studies should assess the utility of such novel markers for in vivo imaging of neuroinflammation, bearing in mind that the binding of second-generation TSPO tracers like PBR28 can be affected by genetic variations (i.e., the rs6971 TSPO polymorphism).^45^

In conclusion, we have provided clear evidence that [^11^C]PK11195 is a sensitive PET ligand for in vivo studies of neuroinflammation in clinical populations with AD and its prodromal stage of amnestic MCI, as well as in a non-AD tauopathy, PSP–Richardson syndrome. The brain regions that showed increased [^11^C]PK11195 binding were those predicted from the well-established pattern of regional cortical and subcortical neurodegeneration in each disease. Our data support the further use of [^11^C]PK11195 PET to study microglia activation in neurodegenerative disorders and in clinical trials that aim to modulate neuroinflammation in neurodegenerative disease.

## Glossary

AD: Alzheimer disease
BP_ND_: nondisplaceable binding potential
CRP: C-reactive protein
ESR: erythrocyte sedimentation rate
MCI: mild cognitive impairment
MMSE: Mini-Mental State Examination
NIHR: National Institute for Health Research
PiB: Pittsburgh compound B
PSP: progressive supranuclear palsy
PSPRS: Progressive Supranuclear Palsy Rating Scale
RAVLT: Rey Auditory Verbal Learning Test
ROI: region of interest
TSPO: translocator protein
